# Characterization of the complete chloroplast genome of *Hedysarum polybotrys* var. *alaschanicum* (Fabaceae) and its phylogeny

**DOI:** 10.1080/23802359.2021.1994900

**Published:** 2021-10-23

**Authors:** Jia-Ning Cao, Chun-Rong Han, Yan-Ci Yang

**Affiliations:** a School of Biological Science and Technology, Baotou Teachers’ College, Baotou, China; b Inner Mongolia Wuhai Wetland Administration, Wuhai, China

**Keywords:** *Hedysarum polybotrys* var. *alaschanicum*, Chloroplast genome, phylogenetic analysis

## Abstract

*Hedysarum polybotrys* var. *alaschanicum* is an important medicinal plant and is widely used in traditional Chinese medicine. The complete chloroplast genome of *H. polybotrys* var. *alaschanicum* was assembled from Illumina pair-end sequence reads. The whole chloroplast genome is 122,933 bp in length and encodes a total of 110 genes, including 76 protein-coding genes, 30 tRNA genes and 4 rRNA genes. The overall GC content of the cp genome is 35.3%. A maximum likelihood (ML) phylogenetic analysis revealed that *H. polybotrys* var. *alaschanicum* was close to *Hedysarum semenovii*.

*Hedysarum polybotrys* Hand.-Mazz. var. *alaschanicum* (B. Fedtsch.) H. C. Fu et Z. Y. Chu, a perennial leguminous herb, is widely used in traditional Chinese medicine to strengthen the body (Hu et al. 2010; Dong et al. 2013). The genus *Hedysarum* L. is economically and medicinally important and widely distributed in the northern hemisphere (Choi and Ohashi 1998). Lots of work has been completed to infer the phylogeny of this genus relying on multilocus (both plastid and nuclear markers) (e.g. Liu et al. 2017; Nafisi et al. 2019), however, the phylogenetic relationship is not well known (Yurkevich et al. 2021). In this study, we report and characterize the complete chloroplast (cp) genome of *H. polybotrys* var. *alaschanicum* (GenBank accession number: MZ327727) to provide genomic data for future research.

An individual of *H. polybotrys* var. *alaschanicum* was collected in Chunkun Mountain, Baotou, Inner Mongolia, China (E110°44′27″,N 41°17′23″). The specimen was deposited at the herbarium of Baotou Teachers’ College (contact person: Yang Yanci, email: yycjyl@163.com) under the voucher number CKS-2020-07-B06KYYHQ. Total genomic DNA was isolated from silica-dried leaf material using modified CTAB method (Doyle 1987), and then sequenced using an Illumina Hiseq 2500 platform at Biomarker Technologies Inc. (Beijing, China). A total of 707,801 reads were assembled to generate the chloroplast (cp) genome of *H. polybotrys* var. *alaschanicum*. Reference-guided assembly was used to reconstruct the cp genomes with the programs MIRA 4.0.2 (Chevreux et al. 2004) and MITObim v1.7 (Hahn et al. 2013). In the process, cp genome of *Hedysarum petrovii* (MT120797) was used as reference genome. The complete cp genome was annotated in GENEIOUS R11 (Biomatters Ltd., Auckland, New Zealand), and then manually corrected by comparing with the published complete cp genomes (GenBank accession numbers: NC_046493, NC_047344) of *Hedysarum* species using GENEIOUS R11.

The complete cp genome of *H. polybotrys* var. *alaschanicum* is 122,933 bp in length. Different from typical quadripartite structure of most angiosperm cp genomes, the cp genome of this species lacks inverted repeat region. The cp genome encodes a total of 110 genes, consisting of 76 protein-coding genes, 30 tRNA genes, and 4 rRNA genes. The overall GC content of the cp genome is 35.3%.

The phylogenetic tree was constructed based on nine complete cp genomes of Fabaceae plants and two complete cp genomes of Rosaceae plants (outgroups). All of the 11 complete cp genome sequences were aligned using MAFFT (Katoh and Standley 2013) with default parameter. To reconstruct the phylogenetic relations of these species we used the GTR + G model with maximum likelihood (ML) analysis. A ML tree was constructed with RAxML v8 (Stamatakis 2014). The branch support values were assessed using a rapid bootstrapping analysis with 1000 replicates. The phylogenetic tree indicated that *H. polybotrys* var. *alaschanicum* was close to *Hedysarum semenovii* with high support (Figure 1). The cp genome of *H. polybotrys* var. *alaschanicum* will provide useful genetic information for further study on genetic diversity and phylogeny of *Hedysarum* species.

**Figure 1.**
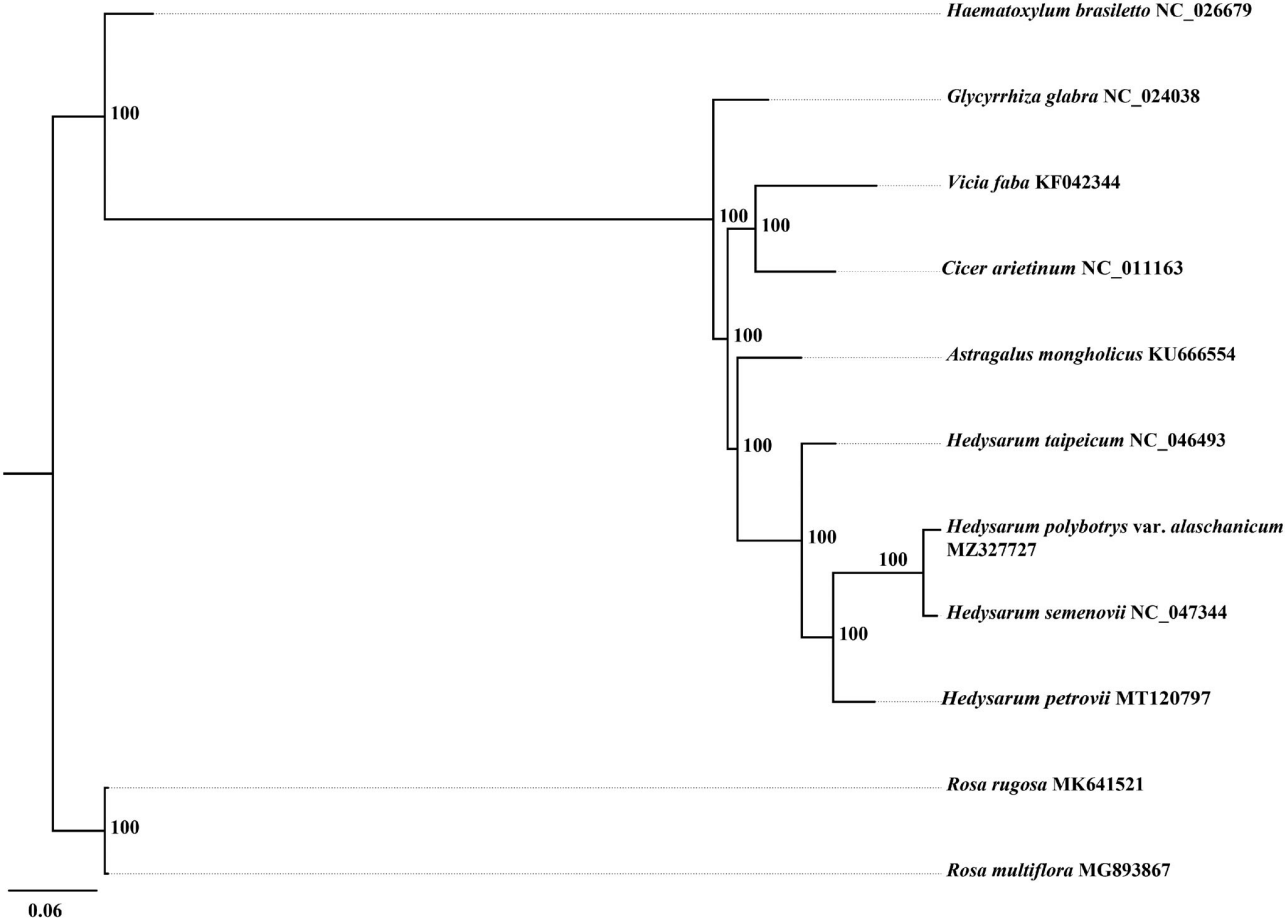
Maximum-likelihood phylogenetic tree for *H. polybotrys* var. *alaschanicum* based on 11 complete chloroplast genome sequences. The number on each node indicates the bootstrap value.

## Data Availability

The genome sequence data that support the findings of this study are openly available in GenBank of NCBI at (https://www.ncbi.nlm.nih.gov/) under the accession no. MZ327727. The associated BioProject, SRA, and Bio-Sample numbers are PRJNA749549, SRP329995, and SAMN20376023, SAMN20376024, respectively.
